# Antibiotics and fecal transfaunation differentially affect microbiota recovery, associations, and antibiotic resistance in lemur guts

**DOI:** 10.1186/s42523-021-00126-z

**Published:** 2021-10-01

**Authors:** Sally L. Bornbusch, Rachel L. Harris, Nicholas M. Grebe, Kimberly Roche, Kristin Dimac-Stohl, Christine M. Drea

**Affiliations:** 1 Department of Evolutionary Anthropology, Duke University, Durham, USA; 2 Program in Computational Biology & Bioinformatics, Duke University, Durham, USA

## Abstract

**Background:**

Antibiotics alter the diversity, structure, and dynamics of host-associated microbial consortia, including via development of antibiotic resistance; however, patterns of recovery from microbial imbalances and methods to mitigate associated negative effects remain poorly understood, particularly outside of human-clinical and model-rodent studies that focus on outcome over process. To improve conceptual understanding of host-microbe symbiosis in more naturalistic contexts, we applied an ecological framework to a non-traditional, strepsirrhine primate model via long-term, multi-faceted study of microbial community structure before, during, and following two experimental manipulations. Specifically, we administered a broad-spectrum antibiotic, either alone or with subsequent fecal transfaunation, to healthy, male ring-tailed lemurs (*Lemur catta*), then used 16S rRNA and shotgun metagenomic sequencing to longitudinally track the diversity, composition, associations, and resistomes of their gut microbiota both within and across baseline, treatment, and recovery phases.

**Results:**

Antibiotic treatment resulted in a drastic decline in microbial diversity and a dramatic alteration in community composition. Whereas microbial diversity recovered rapidly regardless of experimental group, patterns of microbial community composition reflected long-term instability following treatment with antibiotics alone, a pattern that was attenuated by fecal transfaunation. Covariation analysis revealed that certain taxa dominated bacterial associations, representing potential keystone species in lemur gut microbiota. Antibiotic resistance genes, which were universally present, including in lemurs that had never been administered antibiotics, varied across individuals and treatment groups.

**Conclusions:**

Long-term, integrated study post antibiotic-induced microbial imbalance revealed differential, metric-dependent evidence of recovery, with beneficial effects of fecal transfaunation on recovering community composition, and potentially negative consequences to lemur resistomes. Beyond providing new perspectives on the dynamics that govern host-associated communities, particularly in the Anthropocene era, our holistic study in an endangered species is a first step in addressing the recent, interdisciplinary calls for greater integration of microbiome science into animal care and conservation.

**Supplementary Information:**

The online version contains supplementary material available at 10.1186/s42523-021-00126-z.

## Introduction

The long, co-evolutionary history between vertebrates and their microbes underpins the complex web of interactions linking commensal microbiota to host function [1, 2]. Because perturbations to these communities can have both short- and long-term negative consequences [3–5], we increasingly recognize the benefits provided by our endogenous microbiota and have come to view them as ‘old friends’ [6, 7]. To exemplify, while antibiotic treatment effectively combats immediate bacterial infections, it can also lead to prolonged and severe, negative side-effects, such as the elimination of beneficial microbes, increased susceptibility to harmful pathogens [8, 9], and deterioration of microbiome function [10, 11]. Moreover, antibiotics also promote changes in microbial genomes; the ubiquitous use of antibiotics has spurred the spread of genes encoding antibiotic resistance (ABR), which can have potentially catastrophic consequences [12]. Microbial therapies, such as fecal transfaunation, can mitigate the detrimental side-effects of antibiotics [13]; however, because antibiotics are often studied in the context of preexisting illness or injury (which independently influences microbial communities), the severity, duration, and recovery from dysbiosis owing purely to antibiotics remain unclear. Here, we apply an ecological framework in healthy animals to better understand the trajectory and processes governing recovery of or return to a stable, gut microbial community following antibiotic-induced disruption. Because nonhuman primates increasingly serve as models in which to probe microbial dynamics and the development of ABR in response to antibiotic treatment, we experimentally administered a broad-spectrum antibiotic to male ring-tailed lemurs (*Lemur catta*) and used a longitudinal approach to track impacts on the composition and resistomes of their gut microbiota. We further tested the effects of fecal transfaunation as an intervention to promote the recovery of microbial composition and to potentially mitigate the development and persistence of ABR.

Antibiotics and ABR genes have ancient origins as natural compounds or genetic defenses, respectively, used by microbes to compete and survive in densely populated communities, whether within or outside a host [14, 15]. The ability of bacteria to rapidly undergo mutation [16, 17] and share advantageous genes via lateral gene transfer [18, 19] has resulted in myriad, naturally occurring ABR genes [20, 21]. The response of a microbial community to natural antibiotics is largely dictated by the interactions between microbial taxa, which vary over time and across environments. The efficacy and ubiquity of man-made antibiotics have severely perturbed microbial communities via targeted (e.g. narrow spectrum) or indiscriminate (e.g. broad spectrum) elimination of bacterial groups [22], thereby altering the composition and, ultimately, functional potential of microbiomes [23, 24]. In addition, these antibiotics have magnified selective pressure on bacterial communities, making ABR genes advantageous and instigating their proliferation [25, 26], thereby altering the microbiota’s genomic make-up. Within host-associated microbiomes, the propagation of ABR can result in virulent, resistant pathogens [27, 28] that reduce the diversity of native or beneficial microbes [29, 30] and diminish immune capacity of the host. Our understanding of these phenomena primarily derives from studies that characterize the effects of antibiotics on the elimination or development of ABR within specific bacterial pathogens [31, 32]. We know comparatively less about how man-made antibiotics influence the aggregate interactions within presumed healthy, host-associated communities and how those dynamics influence the recovery of microbiota.

Recognizing that commensal consortia are vital to the host has spurred increased research into microbial therapies to mitigate the negative consequences of dysbiosis. In fecal transfaunation, for example, a ‘healthy’ or ‘native’ community of microbes sourced from feces is transferred into a dysbiotic community to combat pathogens and promote the growth of beneficial microbes [33, 34]. Because coprophagy (the ingestion of fecal material either directly or via prey consumption) bolsters gut microbiota during development or illness [35, 36], medical practitioners have examined the use of fecal transfaunations to treat gastrointestinal distress in a wide range of host taxa [37, 38]. As in studies of antibiotics, however, the effects of fecal transfaunation are best understood in the context of infection (with e.g., *Clostridium difficile* [39, 40]). Whether or not fecal transfaunation alters the trajectory of microbiome recovery more broadly remains unclear.

Understudied compared to anthropoid primates, lemurs underwent an unique evolutionary trajectory that makes them particularly diverse and interesting models in which to study the dynamics between hosts and their co-evolved microbes [41–44]. Endemic to Madagascar, the ring-tailed lemur is a diurnal/cathemeral, primarily terrestrial species that lives in multimale-multifemale social groups and shows strict seasonal breeding [45]. Ecologically flexible [45, 46], owing in part to a highly omnivorous diet, it is one of the few lemur species to thrive in captivity. This flexibility is reflected in their resilient gut microbiota that seem relatively unperturbed by aspects of captivity [44]. Ring-tailed lemurs also seem robust to health concerns, such as gastrointestinal problems, that affect the microbiota and welfare of other captive strepsirrhines [47].

Here, we apply classic ecological principles to gut microbial communities to investigate two non-exclusive hypotheses regarding processes of community recovery post perturbation. We experimentally induce perturbation in healthy animals via antibiotic administration, with or without fecal transfaunation. We then pair longitudinal survey of control and experimental animals (during baseline, treatment, and recovery phases) with multiple analytical procedures to examine patterns in microbiota structure (e.g., alpha and beta diversity via 16S rRNA amplicon sequencing), bacterial associations (via Bayesian models of covariation), and ABR gene profiles (via shotgun metagenomic sequencing).

The first hypothesis about recovery is that species diversity improves community stability because it increases functional redundancy [48–50]. Under this ‘diversity begets stability’ hypothesis, as applied to a dysbiotic microbiome, recovery of alpha diversity, regardless of microbial identity, should be vital and sufficient to achieve a stable microbiome [51–53]. Accordingly, after antibiotic treatment, we would expect to see an increase in microbial richness (e.g., alpha diversity), independent of fecal transfaunation. The resulting stable communities of the two treatment groups could thus have similar richness, but different compositions. The second hypothesis is that certain community members (i.e., keystone species or specific ‘old friends’) are foundational to community function [54–57], such that recovery of a stable microbiome requires specific community composition (e.g., beta diversity). Under this ‘keystone species’ hypothesis, we predict that there should be recovery of the same community composition following antibiotic-mediated disturbance, with fecal transfaunation facilitating or accelerating the recovery rate. Accordingly, the resulting stable communities of both treatment groups would eventually have similar compositions.

These two hypotheses could be alternatives or could work in concert, but along different schedules, with potentially more rapid recovery of richness, but slower and more variable recovery of composition. Notably, the complexity of the dynamics between specific community members (i.e., cooperation and competition) could create long-term fluctuations in community composition that would be highlighted by bacterial covariations between key members of the community. Furthermore, the presence of ABR within the microbiomes could exert a distinct force in driving community composition during the treatment and recovery phases. By tracking ABR prevalence and type, coupled with bacterial covariation, we can make inferences about which microbes may be harboring and expressing ABR genes. Our stepwise analytical approach is designed to qualify the short- and long-term impact of antibiotic treatment and test our hypothesis-driven predictions about community recovery.

## Results

### Baseline and control bacterial communities

**Fig. 1:**
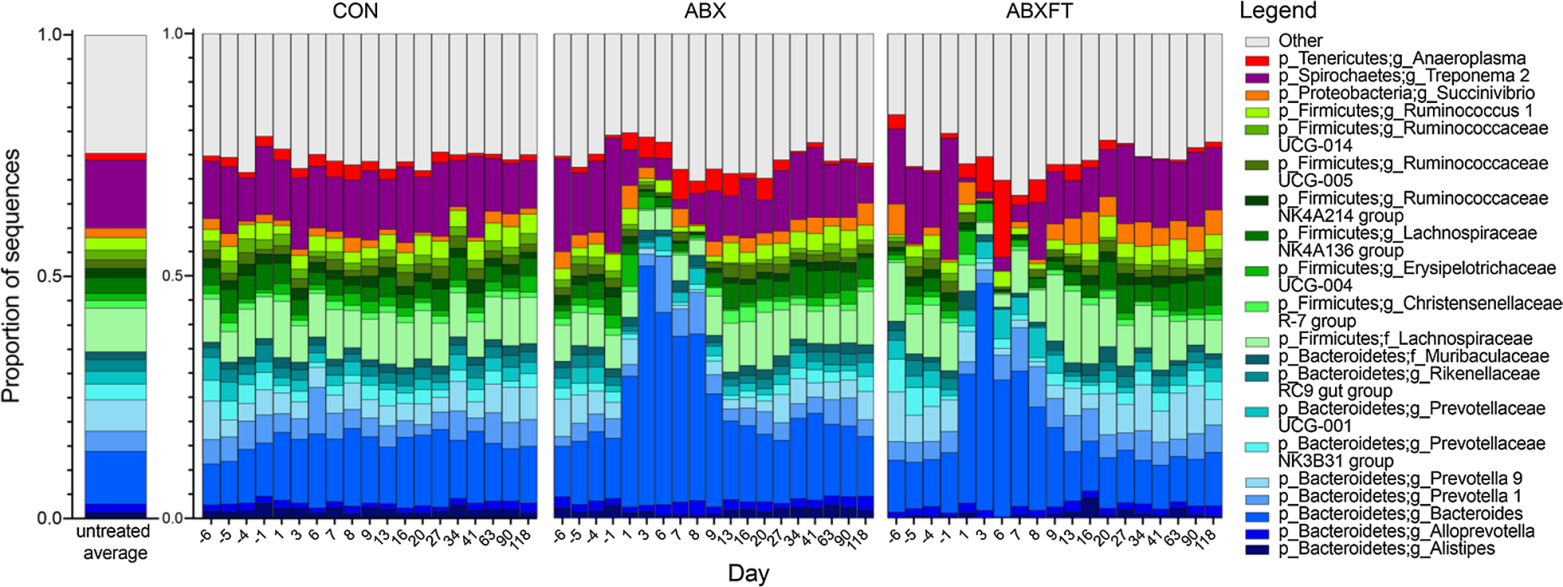
Mean relative abundances of bacterial genera over time in the gut microbiomes of three experimental groups of male ring-tailed lemurs (*Lemur catta*). Shown are values for healthy animals that received no treatment (CON), antibiotics only (ABX), or antibiotics plus fecal transfaunation (ABXFT). Genera are identified by color; those representing < 1% of the microbiomes were combined into the category “Other”. The x axis shows day relative to three phases of study: pretreatment (days -6 to -1), treatment (days 0–6/7), and recovery (days 7/8–118)

**Fig. 2:**
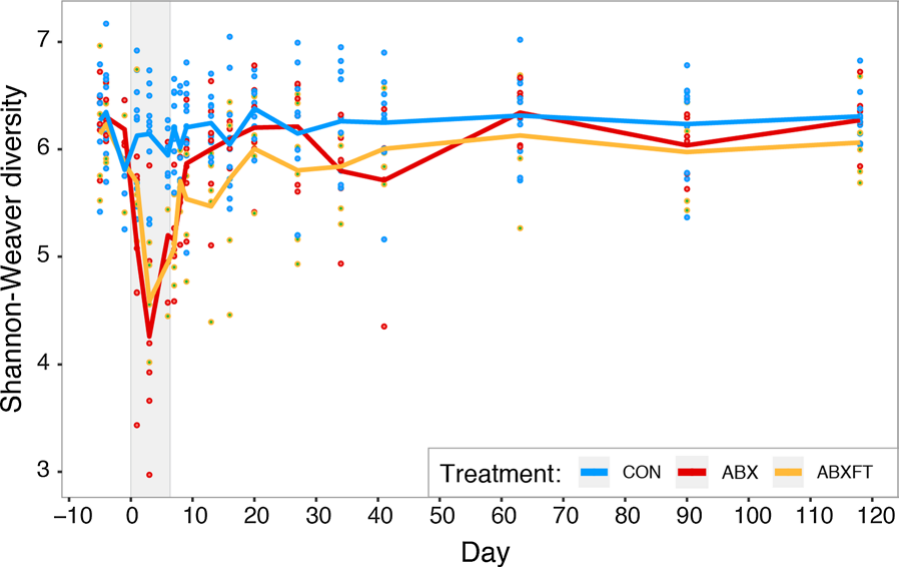
Shannon–Weaver alpha diversity over time in three experimental groups of male ring-tailed lemurs (*Lemur catta*). Shown are values for healthy animals that received no treatment (CON), antibiotics only (ABX), or antibiotics plus fecal transfaunation (ABXFT). Dots represent individual data points and lines connect the mean values of alpha diversity across individuals at each time point. The shaded window represents the period of antibiotic treatment (days 0–6), with fecal transfaunation administered on day 7; all values prior to the onset of treatment represent baseline values and all values post-treatment represent the period of recovery

**Fig. 3:**
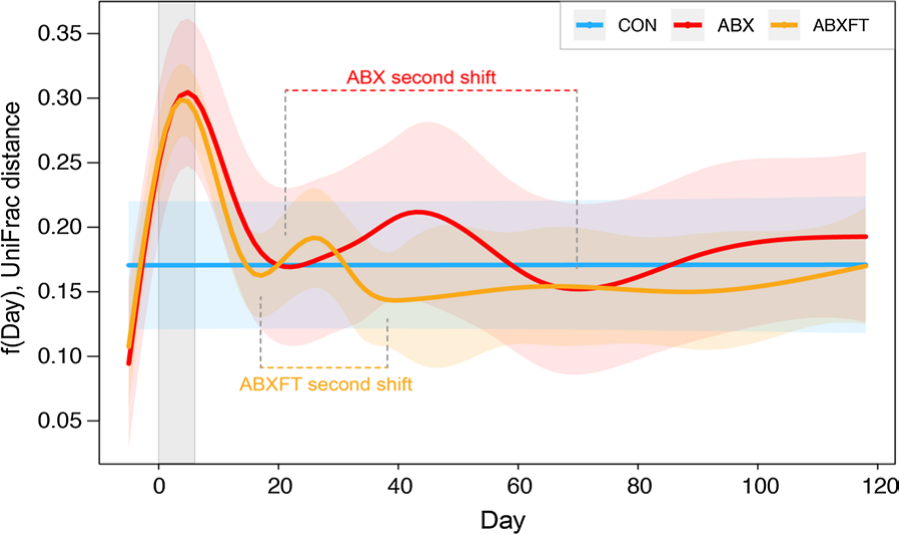
Beta diversity (Unweighted UniFrac distances) for three experimental groups of male ring-tailed lemurs (*Lemur catta*), represented as model-predicted distances from baseline, with 95% confidence intervals. Shown are values for healthy animals that received no treatment (CON), antibiotics only (ABX), or antibiotics plus fecal transfaunation (ABXFT). Trajectories represent predicted responses with smoothing splines that reduce minor variation and noise (e.g., CON animals show minor variation over time in the raw beta diversity data (Figure S1), but the model-predicted values are shown as a straight line). The gray shaded window represents the period of antibiotic treatment, with the prior period representing baseline and the subsequent period representing recovery. The second shifts away from baseline are identified and labelled for ABX and ABXFT animals

Our three animal groups—control (CON), antibiotic-treated (ABX), and antibiotic-treated subsequently receiving fecal transfaunation (ABXFT)—experienced identical baseline or pretreatment conditions, such that we did not expect their gut microbiota to initially differ. Indeed, during baseline, neither alpha nor beta diversity varied significantly between the three experimental groups (alpha diversity: Kruskal–Wallis test, H = 2.478, *p* = 0.289; beta diversity: H = 2.658, *p* = 0.264). Likewise, across all control samples (i.e., across all three phases of study, including pretreatment, treatment, and recovery, for CON animals; n = 184), the dominant bacterial taxa (Fig. 1), as well as the alpha (Fig. 2) and beta (Fig. 3) diversities, remained relatively stable over each of the two years’ four-month study period, showing consistency across the breeding season. Adding the pretreatment phase of the other two groups (baseline samples; n = 43) to the control group (see ‘untreated average’ in Fig. 1), the bacterial gut microbiota of healthy male ring-tailed lemurs, in captivity, were dominated by taxa in the Bacteroidetes and Firmicutes phyla, with lesser contributions from Proteobacteria, Spirochaetes, and Tenericutes. Within these five phyla, 20 genera accounted for minimally 1% of the total sequences (Fig. 1).

### Response to and recovery from antibiotic treatment: alpha diversity and microbial membership

Hereafter, we either combine all three phases of the study (days -6 to 120) so as to present overall differences between the experimental conditions, or we analyze individual phases separately so as to present shorter-term antibiotic effects or specific patterns of recovery. Across the full study, we found a significant effect of experimental group on alpha diversity: relative to CON animals, ABX and ABXFT animals had significantly lower scores (HGAM: CON vs. ABX, t = − 3.535, *p* < 0.001; CON vs. ABXFT, t = − 4.007, *p* < 0.001; Fig. 2). Neither year nor the interaction between year and experimental condition related to bacterial alpha diversity (HGAM1: year, F = 0.001, *p* = 0.990; year*experimental condition, F = 0.942, *p* = 0.391), showing that effects did not differ significantly across the two years of the study.

During the treatment phase only, antibiotic-treated (ABX and ABXFT) animals, relative to CON animals, showed the anticipated and rapid reduction in alpha diversity (days 0–6; HGAM: CON vs. ABX, t = − 4.534, *p* < 0.001; CON vs. ABXFT, t = − 3.754, *p* < 0.001; see shaded bar in Fig. 2). Consistent with the broad effects of amoxicillin, and based on qualitative assessments of relative and log-ratio abundances, antibiotic treatment in healthy lemurs was associated with dramatically reduced microbial representation across a wide range of taxa, including numerous taxa in the Firmicutes phylum, such as members of the Clostridiales class (e.g. Ruminococcaceae and Lachnospiraceae families). Certain taxa, however, were markedly unaffected by antibiotic treatment, including the *Bacteroides* genus and other members of the Bacteroidales family (e.g., *Parabacteroides*), whose log-ratio abundances increased during treatment (See *Bacterial associations* section below for details of log ratio abundances).

During the recovery phase, we found that the differences in alpha diversity between CON and antibiotic-treated animals persisted over the nearly four-month, post-treatment period, suggesting long-lasting imbalance. Compared to CON animals, both antibiotic-treated groups maintained significantly lower alpha diversity (HGAM: CON vs. ABX, t = − 2.256, *p* < 0.025; CON vs. ABXFT, t = − 3.036, *p* < 0.002); however, there were no significant differences between the alpha diversities of ABX and ABXFT animals during recovery (HGAM: ABX vs. ABXFT, t = 0.931, *p* = 0.354; Fig. 2). The latter null finding is inconsistent with fecal transfaunation benefiting recovery of alpha diversity. Lastly, despite their sustained imbalances, both experimental groups exhibited an initial, rapid increase in alpha diversity following antibiotics, consistent with the ‘diversity begets stability’ hypothesis (Fig. 2).

### Response to and recovery from antibiotic treatment: beta diversity

Across all three study phases, we also found experimental condition to be a significant predictor of beta diversity (HGAM2: F = 5.625, *p* = 0.004; Fig. 3), but in a manner that differed from the overall findings on alpha diversity. Notably, compared to CON animals, ABX animals, but not ABXFT animals, showed significantly greater distances from their baseline communities (HGAM: CON vs. ABX, t = 3.434, *p* < 0.001; CON vs. ABXFT, t = 1.726, *p* = 0.085), suggesting greater community recovery with fecal transfaunation; nevertheless, when comparing ABX and ABXFT animals only, again across the entire study period, we found no significant difference in their beta diversity trajectories (HGAM: ABX vs. ABXFT, t = − 1.607, *p* = 0.109).

During the treatment phase, both groups of antibiotic-treated animals showed significantly greater distances from their baseline communities compared to CON animals (HGAM: CON vs. ABX, t =− 3.847, *p* < 0.001; CON vs. ABXFT, t =− 3.761, *p* < 0.001). Given their identical conditions pre-fecal transfaunation, it is unsurprising that the two antibiotic treatment groups did not differ in beta diversity at this time (HGAM: ABX vs. ABXFT, t = 0.081, *p* = 0.935).

During the recovery phase, and when compared to CON animals, ABX animals, but not ABXFT animals, had significantly greater distances from baseline (HGAM: CON vs. ABX, t = 2.790, *p* = 0.005; CON vs. ABXFT, t = 0.599, *p* = 0.549). The greater similarity between the CON and ABXFT groups suggests community recovery consistent with the ‘keystone species’ hypothesis. Furthermore, unlike during the treatment period, when comparing the recoveries of the two antibiotic-treated groups, ABX animals showed significantly greater distance from baseline compared to ABXFT animals (HGAM: t = 2.115, *p* = 0.036; Fig. 3).

Throughout the course of the experiment, the bacterial composition of ABX animals continued to oscillate, whereas in ABXFT animals, bacterial composition became relatively stable approximately 2 weeks after the treatment phase (Fig. 3), consistent with the statistical results reported above. Specifically, after the first compositional shift during the treatment period, the bacterial composition of both ABX and ABXFT animals underwent a second shift away from baseline during the recovery period; however, the magnitude and span of these secondary shifts differed between the ABX and ABXFT groups (Fig. 3). Nevertheless, by the end of the experiment, the beta diversity trajectories of both treatment groups overlapped with those of the control animals, suggesting approximate or incomplete return to baseline.

### Bacterial associations

To characterize the bacterial covariations in the lemurs’ gut, we used pairwise covariation analyses that detected several strong covariations (ρ > 0.5 or ρ < − 0.5; hereafter ‘associations’) between pairs of microbial taxa within the lemurs’ gut microbiomes (Additional file 1: Table S1). We investigated these associations under all three experimental conditions in two stages: across all three phases and during the recovery phase, specifically.

Minimal variation within the microbiota of CON animals limited the detectability of normal bacterial associations; nevertheless, two strong associations emerged. The first was between the genus *Cerasicoccus* and the order WCHB1-41 and was evident across all study phases; the second was between the genus *Cerasicoccus* and the order Rhodospirillales, and was evident during the recovery phase (Additional file 1: Table S1). These two relationships reflect the small-scale, yet ever-present, microbial dynamics that occur in healthy, unperturbed microbiomes.

**Fig. 4:**
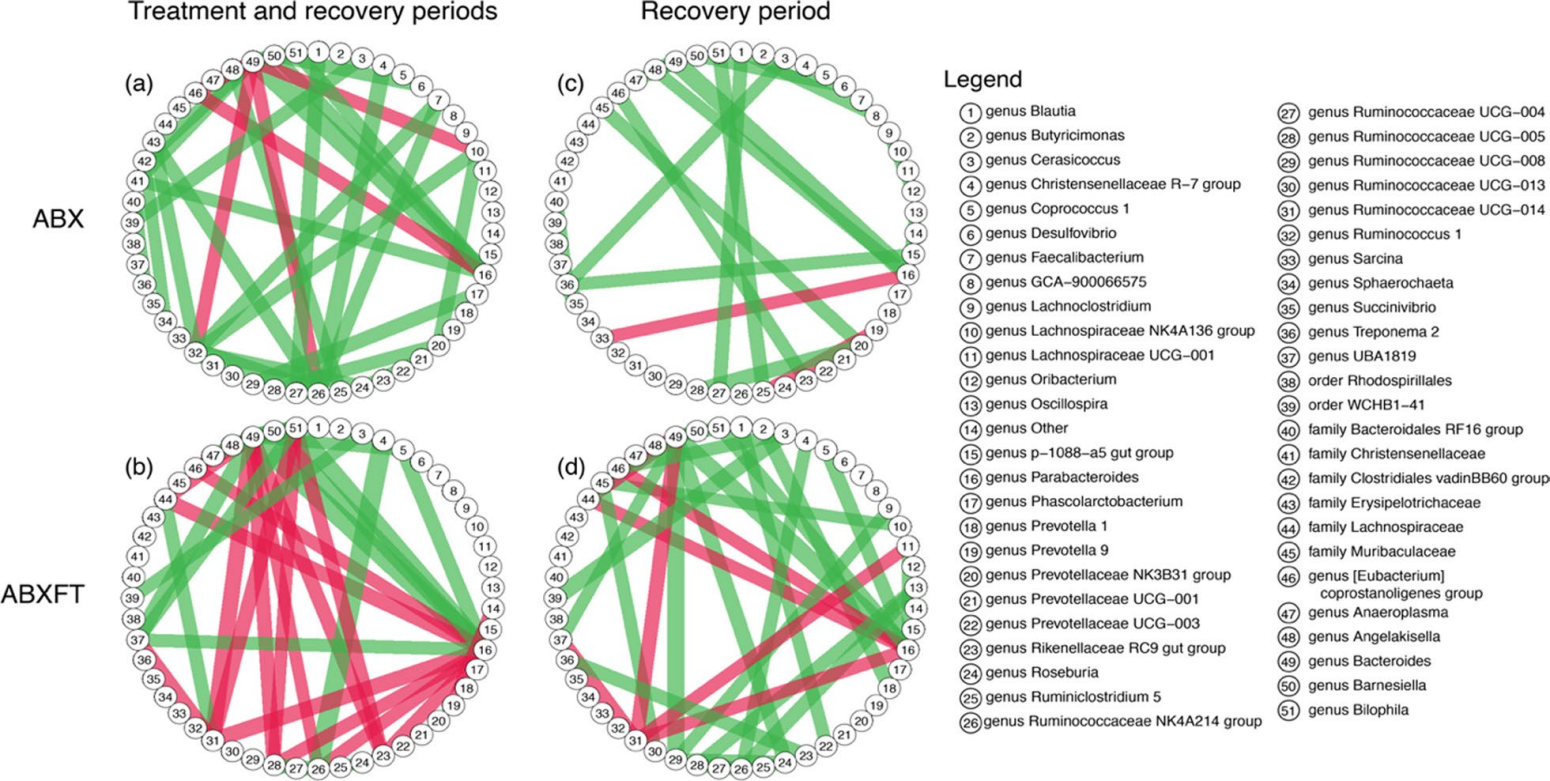
Bacterial associations for healthy, male ring-tailed lemurs (*Lemur catta*) either treated with antibiotics only (ABX) or with antibiotics plus a fecal transfaunation (ABXFT). Line colors represent the direction of the correlation (green = positive, red = negative); line width is scaled to the magnitude of the correlation

**Fig. 5:**
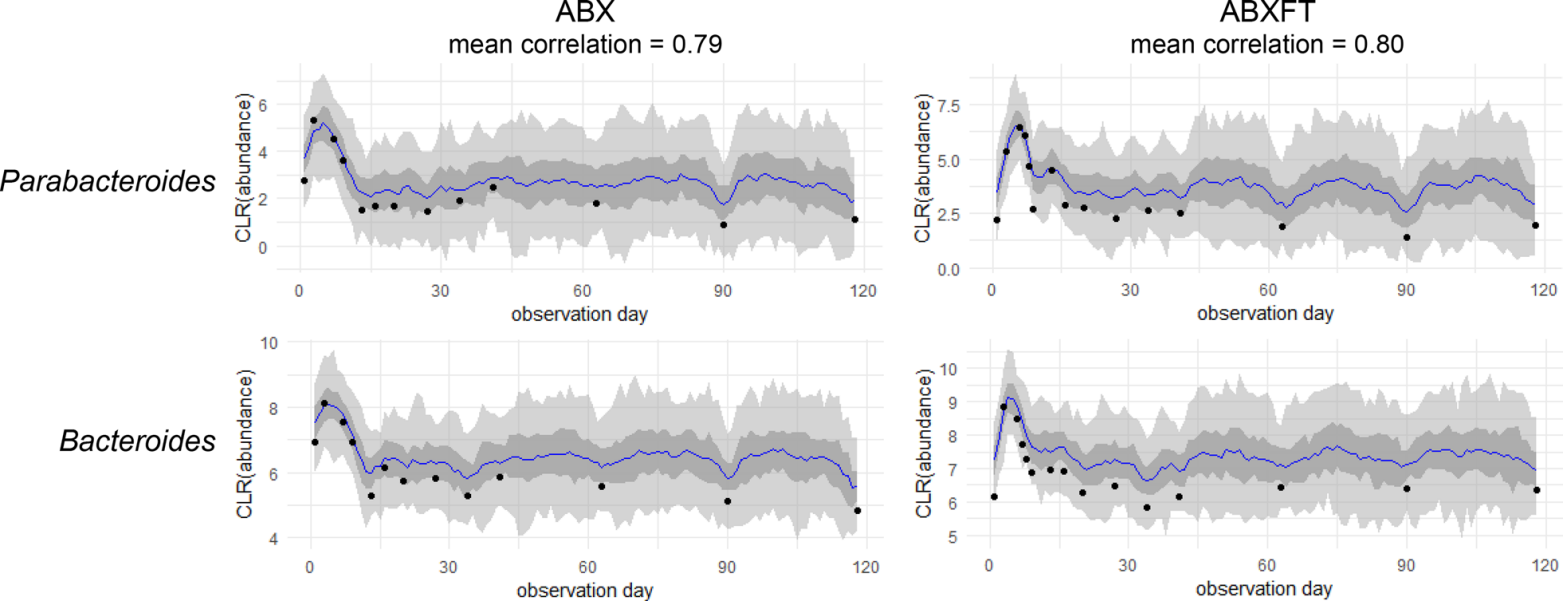
Representative correlation plots for the association between the Centered Log Ratios (CLR; black dots) of *Bacteroides* and *Parabacteroides* abundances in healthy, male ring-tailed lemurs (*Lemur catta*) either treated with antibiotics only (ABX) or with antibiotics plus fecal transfaunation (ABXFT). The blue line is the model-predicted trajectory with the dark and light gray bands representing 50% and 95% posterior predictive intervals, respectively. The antibiotic treatment period spans days 0–6, fecal transfaunation was administered on day 7, and all days thereafter constitute the period of recovery

Within the more variable gut microbiota of ABX and ABXFT lemurs (Fig. 3), and across all three study phases, there were 35 and 31 strong associations, respectively (Fig. 4a, b). In ABX animals, these associations were predominately positive, with only six negative associations, whereas in ABXFT lemurs, positive and negative associations were equally represented (15 and 16, respectively). Shared across ABX and ABXFT animals were 10 strong associations, eight positive and two negative. Within these shared associations, nine involved either *Parabacteroides* or *Bacteroides* (genus 12 and 39, respectively, in Fig. 4a, b). The positive association between these two taxa was the strongest association for both ABX and ABXFT animals (Additional file 1: Table S1). Moreover, in ABX and ABXFT animals, the log ratios of *Parabacteroides* and *Bacteroides* abundances both showed increases during the treatment phase. Because our analyses of log-ratios represent the relationships between abundances of specific taxa relative to the mean abundances of all other taxa, patterns of increasing log-ratios could reflect three possibilities: the abundances of *Parabacteroides* and *Bacteroides* are (1) truly increasing, (2) remaining stable while the mean is decreasing, or (3) decreasing in abundance less dramatically than the mean. Regardless of which pattern they were showing, it would suggest that these two taxa were relatively unaffected by antibiotic treatment (Fig. 5). The majority of strong pairwise associations with *Parabacteroides* or *Bacteroides* were positive, indicating that the associated taxa also withstood the effects of antibiotic treatment, potentially via shared ABR genes.

During the recovery phase, the gut microbiota of ABX animals retained only 20 (18 positive and 2 negative) of their original 35 strong associations (Additional file 1: Table S2, Fig. 4c), likely reflecting the reduction of bacterial taxa that survived antibiotic treatment. By contrast, the gut microbiomes of ABXFT animals retained their same 31 (23 positive and 8 negative) strong associations (Additional file 1: Table S2; Fig. 4d), likely reflecting the immediate reintroduction of baseline microbes via fecal transfaunation. Only two associations were shared between ABX and ABXFT animals during recovery: *Parabacteroides* and *Bacteroides,* and *Christensenellaceae R-7 group* and *Ruminococcaceae NK4A214 group,* both of which were also shared during the entire study period. Despite variability across treatment groups and phases, some of the strongest associations persisted during recovery (Additional file 1: Table S2).

### Cross-sectional and longitudinal ABR

Across the 30 fecal samples selected for shotgun sequencing (from a subset of six subjects), 3.2 million sequences were assigned to 83 known ABR genes. On average, the majority of the ABR genes detected belonged to four resistance gene families: Tetracycline (mean ± SEM; 51.4% ± 3.44%), Beta-lactam (29.5% ± 3.17%), Aminoglycoside (7.9% ± 2.25%), and Macrolide (1.2% ± 0.24%). There was also minor (> 1%) representation of genes in the Vancomycin, Multi-Drug Resistant, and Sulphonamide families.

**Fig. 6:**
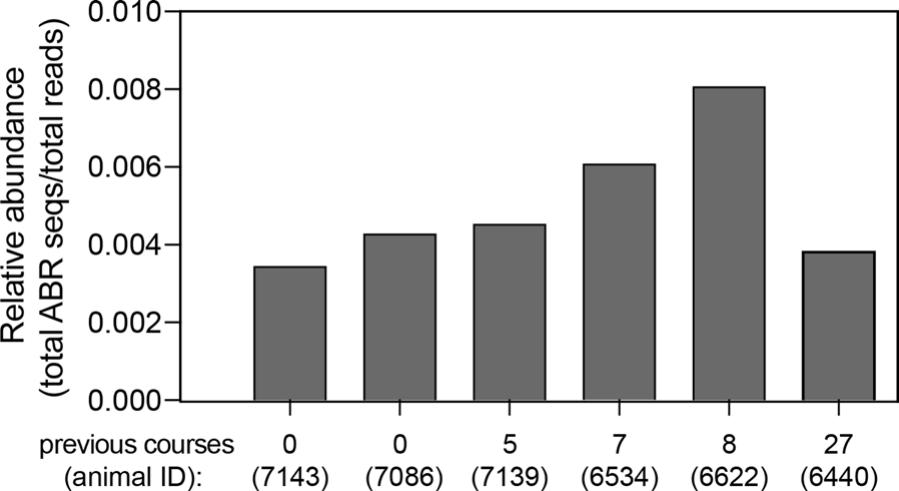
Relative abundance of antibiotic resistance (ABR) genes in six healthy, male ring-tailed lemurs (*Lemur catta*) that had received different numbers of treatment courses of antibiotics across their lifetime

The cross-sectional data on the six, focal animals revealed unexpected variation in ABR. Notably, the two animals (IDs 7143 and 7086) that had never been treated with antibiotics nevertheless harbored ABR at levels similar to those of animals that had previously received numerous courses of antibiotics (Fig. 6). Additionally, the animal (ID 6440) with the most numerous antibiotic treatments (n = 27 courses), harbored the second lowest abundance of ABR genes, similar to that of the animals that had no previous treatment (Fig. 6).

**Fig. 7:**
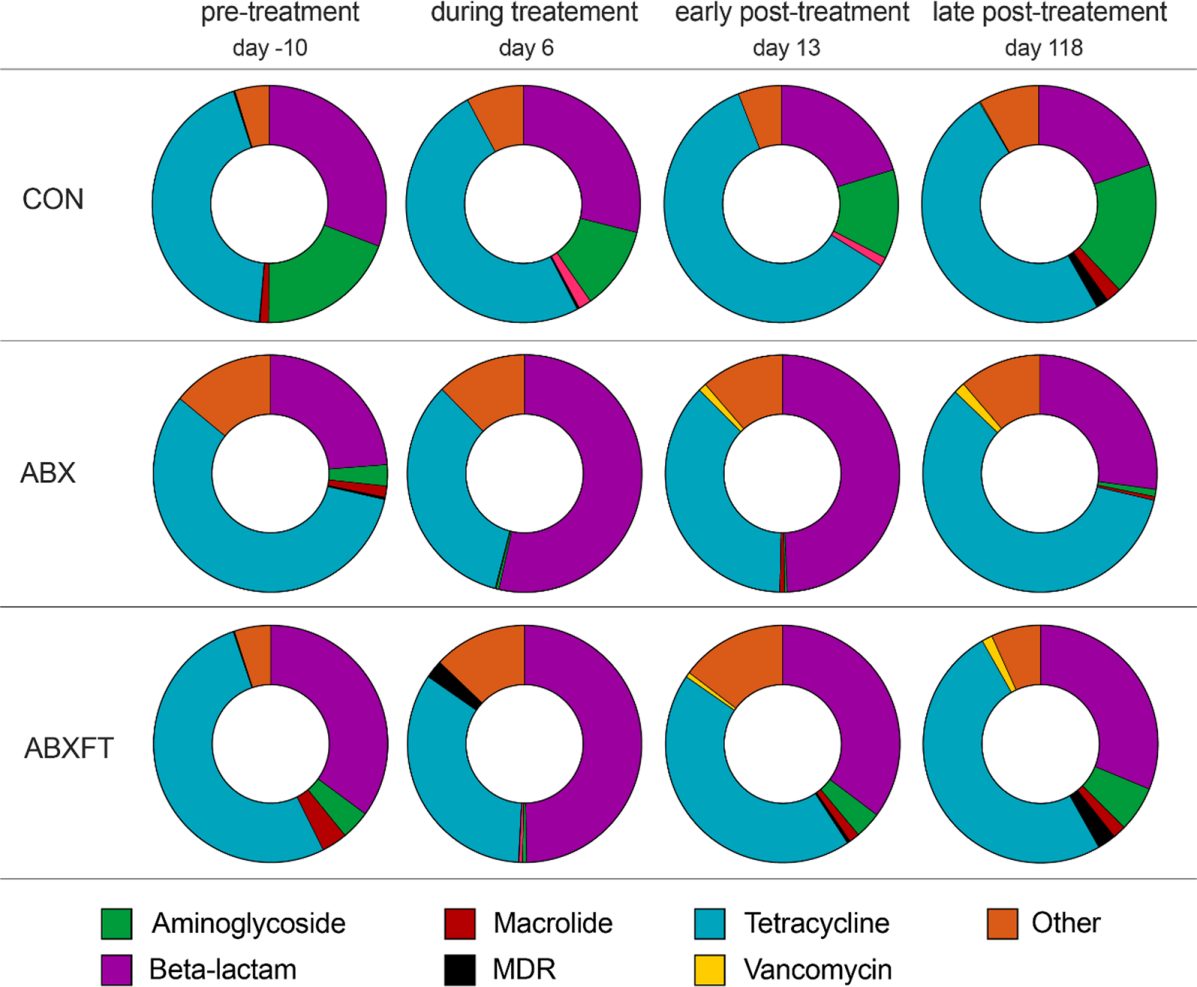
Proportions of antibiotic resistance (ABR) genes identified in healthy, male ring-tailed lemurs (*Lemur catta*) that received no treatment (CON), antibiotics only (ABX), or antibiotics plus fecal transfaunation (ABXFT). Shown are color-coded resistance gene families at four time points during the study, during which antibiotic treatment was administered on days 0–6 and fecal transfaunation was administered on day 7. MDR = Multi-Drug Resistant

**Fig. 8:**
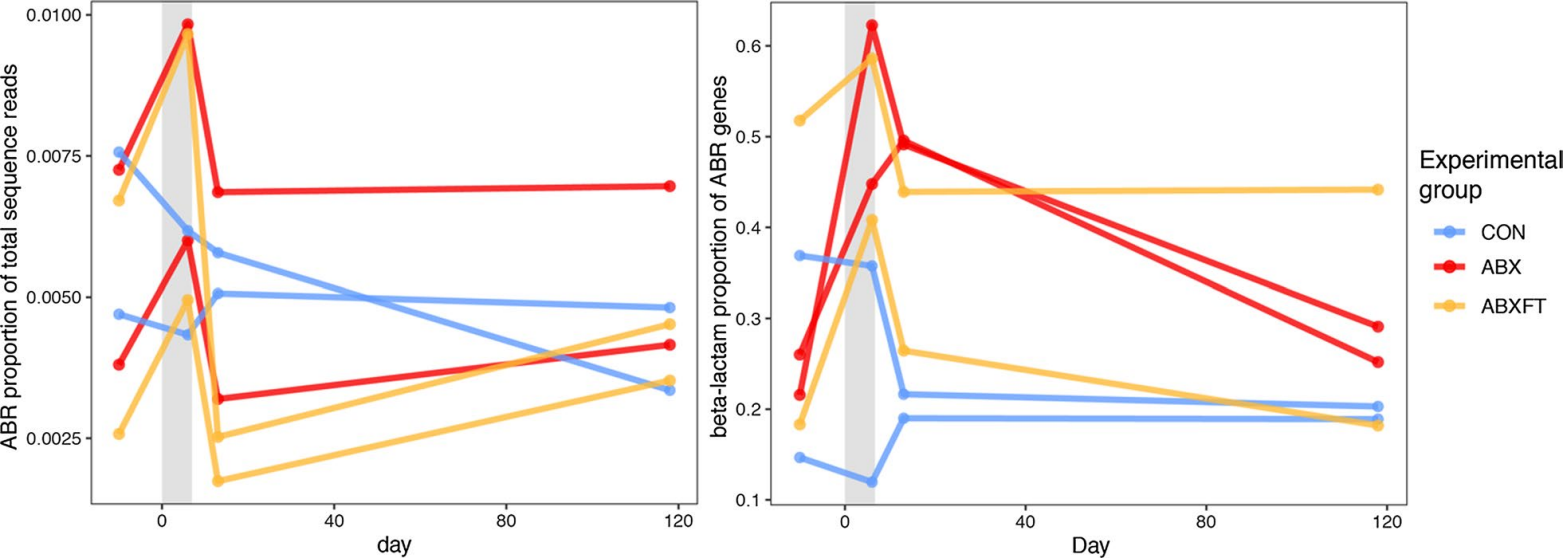
Patterns in antibiotic resistance across healthy, male ring-tailed lemurs (*Lemur catta*) that received no treatment (CON), antibiotics only (ABX), or antibiotics plus fecal transfaunation (ABXFT). Shown is variation over time in **a** the relative abundance of antibiotic resistance (ABR) genes and **b** the proportion of ABR genes assigned to the beta-lactam resistance gene family. The gray shaded window represents the period of antibiotic treatment, with the prior period representing baseline and the subsequent period representing recovery. SEM = standard error of the means

Longitudinally, the relative abundance of ABR genes varied qualitatively across individuals and experimental groups (Figs. 7 and 8). Whereas CON animals showed relatively little variation in mean relative abundance of ABR genes, in both ABX and ABXFT animals, ABR abundance increased between the pre-treatment and treatment phases, followed by a decrease during the recovery period. Similarly, compared to CON animals, ABX and ABXFT animals showed an increase in the proportions of beta-lactam resistance genes during treatment, reflecting the impact of treatment with a beta-lactam antibiotic (amoxicillin; Fig. 8). The proportion of beta-lactam resistance genes during early post-treatment tended to be greater in ABX animals compared to ABXFT animals (Fig. 8), which may hint at a potential mitigating effect of fecal transfaunation on the persistence of ABR in lemur gut microbiomes. Interindividual variation in ABR within treatment groups nevertheless suggests that ABR also may be influenced by factors beyond the scope of this study. Although these results are qualitative, they provide preliminary insights into the acute development and persistence of ABR in relation to antibiotic treatment.

## Discussion

Using a longitudinal, experimental approach in healthy nonhuman primates, we provide support for both the ‘diversity begets stability’ and ‘keystone species’ hypotheses in our ecological framework for interpreting the dynamics of host-associated, gut microbiomes following antibiotic-mediated disruption. This approach allowed us to distinguish different schedules of action associated with rapidly achieving diversity versus slowly recovering keystone species: microbial alpha diversity rebounded quickly (albeit incompletely) in treated animals, whereas beta diversity reflected a trajectory of sustained microbial instability in animals that received antibiotics alone. The effects of fecal transfaunation on recovery of alpha diversity were negligible, but for beta diversity, fecal transfaunation differentiated the recoveries of the two groups receiving antibiotics, providing further evidence that this procedure can hasten and stabilize the recovery of community composition following microbial perturbation. The bacterial associations varied between experimental conditions, reflecting differential sensitivity to antibiotic treatment across bacterial groups and suggesting that microbial dynamics may have contributed to the differential effects of ABX and ABXFT treatments, including through the potential presence of ABR genes. Our cross-sectional analysis of ABR showed that the prevalence of ABR genes in a host is not necessarily correlated with that host’s previous exposure to antibiotics; ABR can be acquired and maintained in the gut microbiomes of lemurs that had no previous antibiotic treatment. Longitudinally, ABR gene profiles varied between individuals and across treatment groups. Qualitatively, the proportion of ABR genes that confer resistance to beta-lactamase antibiotics increased during treatment with amoxicillin. Lastly, fecal transfaunation has the potential to mitigate the persistence of ABR during recovery from antibiotic treatment, a process that warrants further investigation. Using a holistic and longitudinal approach, across scales, allowed elucidating microbial dynamics that otherwise would have been imperceptible.

Consistent with previous findings [58, 59], and with the known efficacy of amoxicillin as a broad-spectrum antibiotic [60], we found that animals receiving antibiotics showed a concurrent and drastic decrease in alpha diversity. Contrary to expectations, however, lemurs post treatment showed a rapid rebound in alpha diversity, with ABX and ABXFT animals showing no significant difference in their recovery trajectories. The rapidity of these patterns may owe to the healthy status of the hosts and to the relatively short period of antibiotic treatment. The ability to recolonize or re-diversify a microbiome after a perturbation can be severely dampened by injury or disease [61, 62] and by recurrent antibiotic treatment [63, 64]. Or, as omnivores that have a broad dietary range, ring-tailed lemurs may have shown more rapid recovery than would be observed by dietary specialists [44]. Beyond external influences (e.g., from diet, the physical environment or social interaction), alpha diversity can also increase from within the host. Indeed, even if antibiotic treatment causes local bacterial extirpations, certain taxa can persist either by expressing ABR genes [10, 65] or by sequestering in areas of the gastrointestinal tract that are less affected by antibiotics (i.e., the appendix or cecum [66, 67]), allowing for in-kind recolonization after disruption. Nevertheless, as evidenced by the results on beta diversity, early recovery of alpha diversity did not entail strictly in-kind recolonization of lemur gut microbiota. The rapidity in these patterns could thus lend support to the ‘diversity begets stability’ hypothesis, in that the key first step in community restoration may be to regain diversity, regardless of microbial composition.

Patterns of beta diversity elucidated longer-term effects of antibiotics on gut microbiome community composition. The relatively unstable community composition of ABX animals suggests that recouping key microbial members may be more elusive (and perhaps more critical to stability) than recouping sheer numbers of taxa. This interpretation is consistent with the ‘keystone species’ hypothesis and with previous evidence [9, 57]. The prolonged absence of key microbial taxa may have significant consequences to the host. Although it was beyond the scope of this study to assess changes in host condition or microbial function, it is well established that antibiotic-induced imbalances in the gut microbiota of healthy animals can cause increased susceptibility to enteric pathogens [8, 9] and altered or diminished immune function [68]. Likewise, even natural variation in microbial community composition (e.g., between seasons or host populations) are associated with changes in microbiome function [69–72].

Patterns of beta diversity also revealed the potential stabilizing effect on community composition of fecal transfaunation, again consistent with the ‘keystone species’ hypothesis. In our study, hosts were administered their own baseline feces, but similar findings were obtained in a previous study in which antibiotic-treated mice consumed feces from healthy cagemates [73]. The bacterial associations that characterized the recovery phase in ABXFT lemurs were more numerous and almost wholly different from those in ABX lemurs, indicating that microbial interactions may underpin some of the effects of fecal transfaunation. These findings are consistent with the concept of competitive exclusion, whereby the diverse group of reintroduced, native bacteria outcompete pathogenic or opportunistic microbes [74, 75]. Although there is much to learn about the modes of action in successful transfaunation, we contribute evidence, unconfounded by host health status, for this promising tool to hasten recovery from antibiotic exposure [76–78]. That statistical differences between the ABX and ABXFT trajectories were associated with examining different portions of the study may reveal that an exceptionally strong, initial impact of antibiotic treatment outweighs other, potentially more subtle, effects on community structure. Detecting the latter may require examining post-perturbation recovery over longer periods than have been traditionally adopted.

Of the bacterial associations present in the two treatment conditions, *Parabacteroides* and *Bacteroides*—two, closely related taxa with similar functional potential [79]—dominated the observed relationships. Notably, increases in the log ratio of *Bacteroides* during antibiotic treatment indicated that, while other taxa were eliminated, abundances of *Bacteroides* members were increasing, remaining stable, or decreasing less dramatically relative to the mean abundances of the other taxa. Indeed, the *Bacteroides* genus is notorious for showing ABR. The diversity of its resistance mechanisms [80, 81], coupled with extensive lateral gene transfer within members of the genus and with non-*Bacteroides* taxa [82, 83], contributed to certain *Bacteroides spp*. having one of the highest resistance rates among known anaerobic pathogens [84]. Furthermore, certain *Bacteroides* species can harbor an unknown or potentially diet-mediated mechanism that confers resistance specifically to amoxicillin [85, 86]. Certain *Bacteroides* strains can even shield other taxa from the effects of beta-lactam antibiotics [87]. Combined with this evidence, our results suggest that *Bacteroides* in the lemur gut microbiome likely have amoxicillin resistance mechanisms and that bacterial taxa with log-ratios that similarly increased during treatment may share similar resistance to amoxicillin treatment.

ABR genes, including some that are considered clinically relevant [88], were present within the gut microbiome of all lemurs. Somewhat surprisingly, lemurs that had never received antibiotic treatment showed abundances of ABR similar to those of lemurs previously treated with antibiotics. Researchers have shown that bacteria and their genes can be shared between hosts that cohabitate [89, 90] or share social partners [91]. Furthermore, ABR genes often reside on mobile genetic units and are prone to rapid transfer between microbes [92]. Indeed, the ‘resistance crisis’ is perpetuated by the ubiquitous spread of ABR genes around the world [12, 93, 94]. Here, we find that lemurs are not exempt from these phenomena and, for captive animals especially, ABR could pose a severe threat to animal health [95, 96]. Methods to mitigate the development and spread of ABR among animal populations, including perhaps via fecal transfaunation, may prove to be a critical facet of combatting the resistance crisis [93, 97].

## Conclusions

Collectively, these results further our understanding of host-microbe relationships in the Anthropocene era [98, 99]. Because antibiotics are an unavoidable component of animal care, understanding their impact on host-associated communities will provide context for studying the biology of animals under human care and strengthen protocols for animal well-being. Ultimately, shedding light on how ‘old friends’ react to aspects of the ‘new world’ is relevant both to our understanding of the evolution of symbiosis and to its implications for animal welfare and conservation.

## Methods

### Study subjects and housing

Our study subjects were 11 healthy, reproductively intact, adult (4–16 years old), male ring-tailed lemurs housed in 10 conspecific, mixed-sex groups at the Duke Lemur Center (DLC; Durham, NC, USA). Within a two-year period, 10 subjects underwent a control round with no treatment, but all 11 underwent one round of antibiotic treatment (see below), all while living in their same social groups. During inclement weather (< 5 °C), the groups would be sequestered in temperature-controlled indoor enclosures, otherwise, they all had access to indoor and outdoor enclosures (approximately 146 m^2^/animal). Some of the groups additionally had access to large, forest enclosures where they semi-free-ranged with heterospecific lemurs. The animals received a diet of produce and commercial primate chow and, while semi-free-ranging, had access to natural foods foraged from the forest. Additional information on the lemurs’ diet, foraging, and social behavior have been reported elsewhere [100]. The subjects were maintained in accordance with the NIH Guide for the Care and Use of Laboratory Animals, and procedures were approved by the Institutional Animal Care and Use Committee of Duke University (protocol #A111-16-05).

### Study design and sample collection

To allow for a partial cross-over design, we conducted the study during two matched periods (October-February) in consecutive years, during the subjects’ breeding season in the Northern Hemisphere [101]: 2016–2017 (Y1, n = 10 subjects) and 2017–2018 (Y2, n = 11 subjects). In each year, we assigned the subjects to one of three experimental groups: control animals (CON: Y1, n = 4; Y2, n = 6), antibiotic-treated animals (ABX: Y1, n = 3; Y2, n = 3), and antibiotic-treated animals receiving a fecal transfaunation (ABXFT: Y1, n = 3; Y2, n = 2). To avoid administering antibiotics twice to any animal, each animal was assigned to the CON group in one of the two years.

Each year of study involved three phases, lasting a total of ~ 125 days: a pretreatment or baseline phase (lasting ~ 6 days; i.e., days -6 to -1), a treatment phase (lasting 7–8 days; i.e., days 0 to 6/7), and a recovery phase (lasting ~ 110 days). In the treatment phase, all treated animals (ABX and ABXFT; n = 11) received a 7-day course of the broad-spectrum, beta-lactam antibiotic, amoxicillin (10 mg/kg body weight, received orally, twice daily). Approximately 24 h after completion of the full antibiotic regimen, ABXFT subjects received a fecal transfaunation consisting of their own feces collected pretreatment (4 days prior to the onset of treatment for all animals): 2–3 fecal pellets were mixed with water and administered orally via syringe or feeding tube, according to routine procedures that have been adopted by the DLC since the mid 1980s to treat outbreaks of gastrointestinal diseases [47, 102, 103].

The study phases were additionally differentiated by the frequency with which we collected fecal samples: We collected samples every 1–3 days before, during, and immediately after the treatment phase, after which sampling occurred every 5–28 days. Typically, upon the subject’s morning voiding, between 7:00 am and 11:30 am, we opportunistically collected fresh fecal samples. On occasion, we collected samples from awake, gently restrained animals that were habituated to capture and collection procedures. At each time point, we sampled all subjects and we maintained analogous sampling regimes across years. We collected all samples in sterile, 15-ml falcon tubes, immediately placed them on ice, and then stored them in a − 80 °C freezer within 2 h, until analysis.

### Microbial DNA extraction, sequencing and bioinformatics

Using the DNeasy Powersoil kit (QIAGAN, Frederick, MD), we extracted microbial gDNA from fecal samples and from four blank controls, to control for possible contamination. We quantified the extracted gDNA using a Fluorometer (broad-spectrum kit, Qubit 4, Thermo Fisher Scientific, Waltham, MA). These extractions were used for bacterial identification (via 16S rRNA amplicon sequencing) and ABR gene identification (via shotgun sequencing), as described below.

### Bacterial identification

We shipped aliquots of extracted gDNA to the Argonne National Laboratory’s Environmental Sequencing facility (Lemont, IL) for library preparation and sequencing of the 16S rRNA gene. There, the V4 region of the 16S rRNA gene (515F-806R) was amplified with region-specific primers adapted for the Illumina MiSeq platform [104]. Forward primers contained a 12-base barcode sequence to support pooling of samples in each flow cell lane. Once pooled, amplicon libraries were cleaned using AMPure XP Beads (Beckman Coulter, Pasadena, CA), and quantified using a fluorometer (Qubit 4). Amplicons were sequenced on a 2 × 151 bp Illumina MiSeq run [104]. Sequencing reads are available on the National Center for Biotechnology Information's Sequence Read Archive (BioProject ID PRJNA765714).

In collaboration with Duke University’s Genomic Analysis and Bioinformatics Shared Resource, 16S raw sequence data were analyzed using a bioinformatics pipeline generated in QIIME2 (ver 2018.11) [105]. The pipeline included steps to join, demultiplex, and quality-filter sequence reads. The DADA2 plugin (q2-dada2) [106] was used to denoise, quality-filter, and remove phiX and chimeric sequences from the demultiplexed reads. Using the resulting sequences, we discarded samples with < 10,000 reads. To determine taxonomic classification, we used a pre-trained Naive Bayes classifier at 99% sequence identity (SILVA-132 database) [107, 108]. After bioinformatic processing, a total of 344 fecal samples (from all subjects across all study phases) yielded over 23.4 million 16S sequences (mean per sample = 59,766).

To calculate metrics of alpha and beta diversity, we first subsampled our data to a depth of 15,000 reads per sample. We then used the feature tables and taxonomies of bacterial members to calculate Shannon–Weaver diversity (i.e., alpha diversity). To assess microbial composition (i.e., beta diversity), we calculated multiple metrics of UniFrac distances. Both UniFrac metrics showed similar patterns of microbial composition (Additional file 1: Figures S2 & S3), and so, in our results, we report only unweighted UniFrac, which considers the phylogenetic relationships between taxa and, importantly, gives equal weight to rare and abundant taxa. After calculating these diversity metrics, we combined features without assigned taxonomy below the Kingdom level into an “Unassigned” category. We also included the conglomerate “Other” to represent the rare taxa that had relative abundances < 1%.

### ABR identification

To allow for cross-sectional and longitudinal analyses of ABR genes, while limiting the expense of metagenomic analyses, we performed shotgun sequencing on samples from six lemurs, two per experimental group, that ranged in their previous exposure to antibiotics (0–27 previous courses). For cross-sectional analysis, we included one sample from each subject’s pretreatment phase in Y1. For longitudinal analysis (which we prioritized), we included four samples from each animal (days -10, 6, 13, and 118) in Y2. We shipped this subset of extractions (n = 30) for shotgun sequencing to CosmosID (Rockville, MD), where DNA libraries were prepared using the Illumina Nextera XT library preparation kit, with a modified protocol. Library quantity was assessed with Qubit (ThermoFisher). Libraries were then sequenced on an Illumina HiSeq platform 2 × 150 bp.

The samples selected for shotgun sequencing averaged 20.4 million sequences per sample. The resulting unassembled sequencing reads underwent multi-kingdom microbiome analysis and profiling of antibiotic resistance genes using the CosmosID bioinformatics platform (CosmosID Inc., Rockville, MD), as described elsewhere [109, 110]. The antibiotic resistance and virulence genes in the microbiome were identified by querying the unassembled sequence reads against the CosmosID-curated antibiotic resistance and virulence associated gene databases [111, 112].

### Statistical analyses

To characterize variation in bacterial diversity and composition, we used Hierarchical General Additive Models (HGAM) [113], which have the flexibility to accommodate nonlinear trends (for full model syntax and model description, see Additional file 1, *i. Descriptions of statistical models*). HGAMs use predictor and response variables to predict smooth functional relationships that can vary by different groups (e.g., the three experimental groups). In our case, the three experimental groups were expected to have different response trajectories, so our HGAMs were structured to fit smoothing splines specific to the responses of each experimental group over time. We used this model to test for patterns in bacterial alpha and beta diversity. For analyses of beta diversity, we first used Principal Coordinates Analysis (PCoA) to visualize variation in bacterial composition (both unweighted and weighted UniFrac distances) in coordinate space (Additional file 1: Figures S2 and S3, respectively). Subsequently, we used distance metrics to calculate change in bacterial composition relative to a pretreatment, baseline community (these were the samples used for FT, collected 4 days before the onset of treatment for all animals; QIIME2). We tested for variation in these calculated distance measures using our HGAM. To assess the overall trajectories of the diversity metrics, and those during specific experimental periods, we used our models in a stepwise approach that allowed us to test for variation across the entire dataset and in each of the three periods.

To better understand the short- and longer-term process of recovery, we tested for associations between bacterial taxa over time and evaluated how these associations may have differed between treatment groups. To exclude spurious associations, we first removed the pretreatment samples from each animal's series, further allowing us to focus on associations during the treatment and recovery phases. To reduce sparsity in the dataset and ease the computational burden, we removed rare taxa present in less than five samples across the full dataset, clustered taxa at the genus level, and grouped as ‘Other’ all low-abundance genera with less than 0.01% of total counts. This filtering removed less than 1% of total sequence counts.

To naturally model the irregular temporal spacing in the observations and manage autocorrelation between samples, we fitted a Bayesian multivariate Gaussian process to each of multiple, synthetic replicates of the dataset (see resampling procedure below and a detailed description of this procedure in Additional file 1, *i. Descriptions of statistical models*). We then inferred a distribution over the covariance between microbes. The sample collection schedule motivated two key choices in noise modeling and data representation. First, because stochasticity exists in sample collection, processing, and sequencing, we used a resampling method similar to that of ALDEx2 [114, 115] to emulate the variation that would be expected from replicate measurements. Second, to account for the compositional nature of the sequence count data within our model [116], we used sequence counts to calculate log ratio abundances that reflect the relationship between the abundances of specific taxa and the geometric mean of all other taxa within the community at a given time point. We converted the estimated covariance matrices to correlation matrices and thresholded all pairwise correlations between microbes to select as significant those with 95% credible intervals that excluded zero correlation (i.e., those with strong positive or negative associations). We then ranked associations by their median strength and selected those in excess of correlation ρ > 0.5 or ρ < − 0.5 as strong associations.

Because both our cross-sectional and longitudinal ABR data had small sample sizes and minimal statistical power, we were limited to examining qualitative trends in the prevalence of total ABR genes and the types of ABR gene family to which they belonged.

## Supplementary Information

**Additional file 1.** Supplementary file with expanded description of statisical models, supplementary figures, and supplementary tables.

## Data Availability

Sequencing reads are available in the National Center for Biotechnology Information's Sequence Read Archive (BioProject ID PRJNA765714). Additional datasets on antibiotic resistance genes, along with specific figures adjusted to be discernible to colorblind readers, are available in Open Science Framework (https://osf.io/3gf5t/). Further genomic data are available from the corresponding authors upon reasonable request.
