# Supplementary material for: Antibiotics and fecal transfaunation differentially affect microbiota recovery, associations, and antibiotic resistance in lemur guts
Source: Anim Microbiome. 2021 Oct 1;3:65. doi: 10.1186/s42523-021-00126-z (PMC8485508; doi:10.1186/s42523-021-00126-z)
Supplement: Supplementary file 1 — Additional file 1. Supplementary file with expanded description of statisical models, supplementary figures, and supplementary tables. [file 42523_2021_126_MOESM1_ESM.docx]

**Supplementary Materials**

Antibiotics and fecal transfaunation differentially affect microbiota recovery, associations, and antibiotic resistance in lemur guts

Sally L. Bornbusch, Rachel L. Harris, Nicholas M. Grebe, Kimberly Roche, Kristin Dimac-Stohl, Christine M. Drea

***i. Descriptions of statistical models***

Hierarchical Generalized Additive Models (HGAMs): We used HGAMs to test for variation in the trajectories of alpha and beta diversity, over time, between the three experimental groups of lemurs: CON, ABX, and ABXFT. All models are structured following Pedersen *et al*., 2019 and we report model syntax for use in the R (ver. 4.0.2) via the gam() function in package {mgcv}. The models were run on data spanning the entire experiment and on subsets of data spanning the treatment period and/or recovery period. The model for testing variation in alpha and beta diversity was as follows:

Full Model: Diversity_metric ~ Experimental_group * Year + s(Day, by = Experimental_group) + s(Animal, by = Year, bs = "re") + s(Experimental_group, bs = "re"), method = “REML”

Model term explanations:

Experimental_group * Year: Testing for fixed effects of experimental group, year, and the interaction between the two.

s(Day, by = Experimental_group): provides a group-specific smoothing spline for each experimental group that accounts for differences in response trajectory over time (Day).

s(Experimental_group, bs = "re"): because group-specific intercepts are not incorporated into factor-by-variable smoothers (e.g., the term described above), this term represents random effects for the intercepts (bs="re" term) of the different experimental groups.

s(Animal, by = Year, bs = "re"): similar to the above term, this term provides random effects for the intercepts of each animal with a smoothing spline that is specific to the year (to account for different patterns of the same animal across the two different years).

Bayesian multivariate Gaussian process regression: We used this statistical method to test for covariation between the log-ratios of microbial taxa. Log ratios reflect the abundance of specific taxa relative to the mean abundance of all other taxa, thus negating concerns on the compositional nature of microbiome data. Counts in each sample were resampled in a procedure similar to that performed by Fernandes et al. (2014). This procedure was repeated 500 times, yielding 500 resampled instances of the original data set with bacterial sequence variants rendered as log-ratios.

Per-cohort observation matrices were constructed by aggregating samples within a cohort (i.e. CON, ABX, ABXFT) to give one such observation matrix Y per cohort per resampling instance. We independently fit each of these resampled instances using the following Bayesian multivariate Gaussian process regression model:

Here X is vector of sample times indices (days).

Line (1) describes the deviation in observed values from their moving averages (latent parameter Λ). Parameter Σ describes the covariation between log-ratio sequence variants.

Line (2) describes the deviation in the moving averages from their baseline values (specified by Θ). Again, Σ mediates covariation between log-ratio sequence variants. Parameter Γ is a kernel matrix that encodes (autoregressive) covariance between samples.

Line (3) specifies a prior belief about the scale and structure of the log-ratio covariance matrix Σ. Parameters for this prior were chosen to be effectively non-informative.

This model fits a smooth trajectory, varying around Θ, to each log-ratio, enforcing the between-sample correlation specified by Γ. Inference on the remaining parameters, Λ and Σ, can be performed by treating this model as an instance of Bayesian multivariate linear regression, which gives closed-form estimates for the posterior (data-informed) distributions of Λ and Σ.

For each resampled instance of the data set, we calculated the *maximum a posteriori* estimate for Σ, yielding a single, most probable estimate for the covariance across log-ratio sequence variants given a single resampled instance of the data set.

***ii. Supplementary figures of beta diversity (Figures S1, S2, and S3)***

Figures S1, S2, and S3 show beta diversity (i.e., community composition) for all subjects (CON, ABX, and ABFT).

In the main text we present the model-predicted values of unweighted UniFrac distances (Figure 3). In Figure S1, we present the raw beta diversity data for unweighted UniFrac. These data reflect the same patterns seen in Figure 3 and demonstrate the minimal variation seen in CON animals.


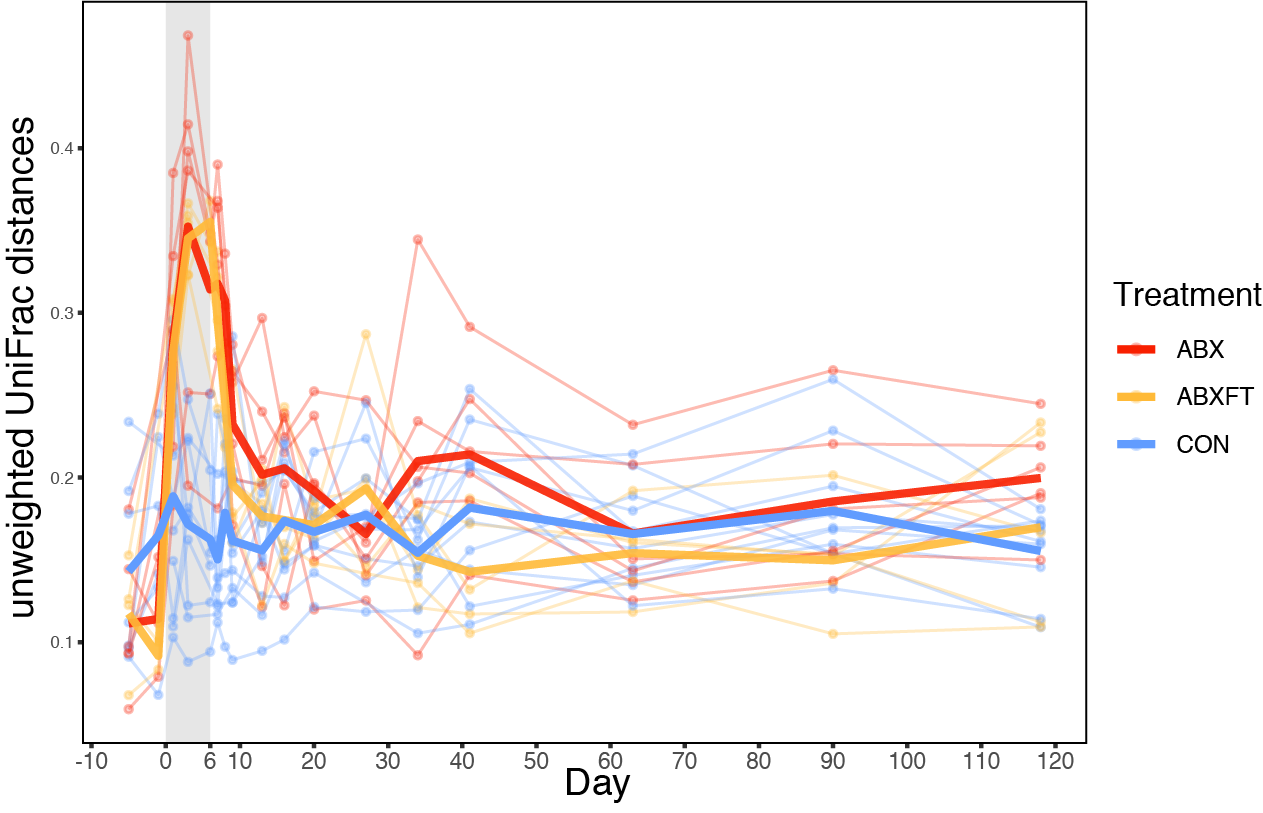


Figure S1. Figure S1. Change in bacterial composition (Unweighted UniFrac beta diversity) over time relative to a pretreatment, baseline community (collected 4 days before the onset of treatment for all animals and the same samples used for FT). Shown are the values for healthy, male ring-tailed lemurs (*Lemur catta*) that received no treatment (CON), antibiotics only (ABX), or antibiotics plus fecal transfaunation (ABXFT). Dots represent individual data points and lines connect the values for each animal over time. The bolded lines represent mean values of alpha diversity for each experimental group. The shaded window represents the period of antibiotic treatment (days 0-6), with fecal transfaunation administered on day 7; all values prior to the onset of treatment represent baseline values and all values post-treatment represent the period of recovery.

In figures S2 and S3, we present principal coordinate plots of unweighted and weighted UniFrac diversity, respectively. In each figure, the top plot (a) shows all data points colored by experimental group. The three plots below (b, c, d) are the data from a representative individual in each of the three experimental groups. These figures show that, for both metrics of beta diversity, the microbiota of CON animals vary little over time, whereas the animals in both treatment groups experience a large shift in gut microbiota composition (to the right of the plot) associated with antibiotic treatment, followed by recovery of community composition akin to
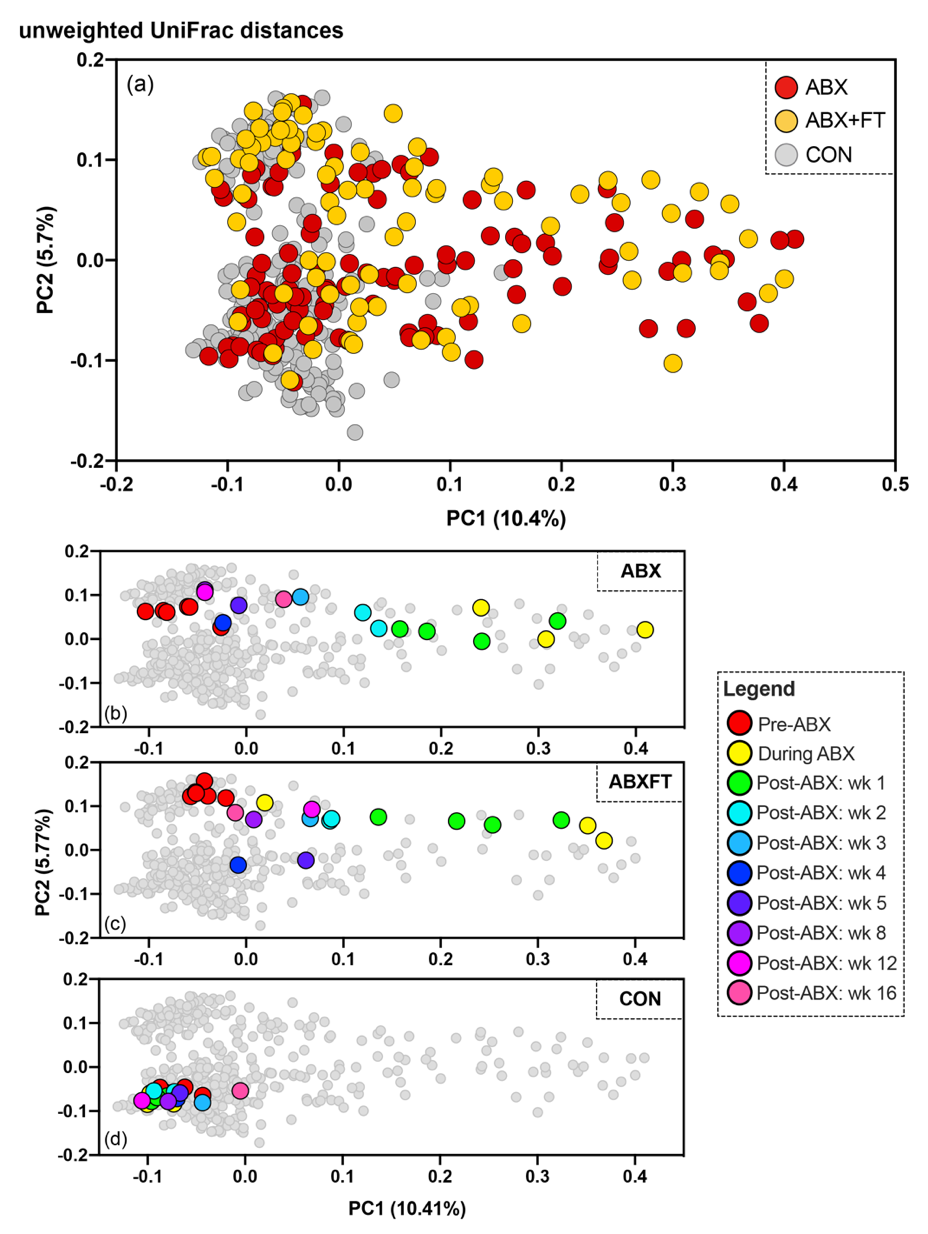
their pre-treatment microbiota (the left of the plot).

Figure S2. Principal coordinate analyses of unweighted UniFrac beta diversity including (a) all samples colored by the three experimental groups: healthy, male ring-tailed lemurs (*Lemur catta*) that received no treatment (CON), antibiotics only (ABX), or antibiotics plus fecal transfaunation (ABXFT). (b, c, d) beta diversity of a representative animal from each of the three experiment groups with data points colored according time of collection (pre-, during, and post-antibiotic treatment; gray points are data from all other animals).


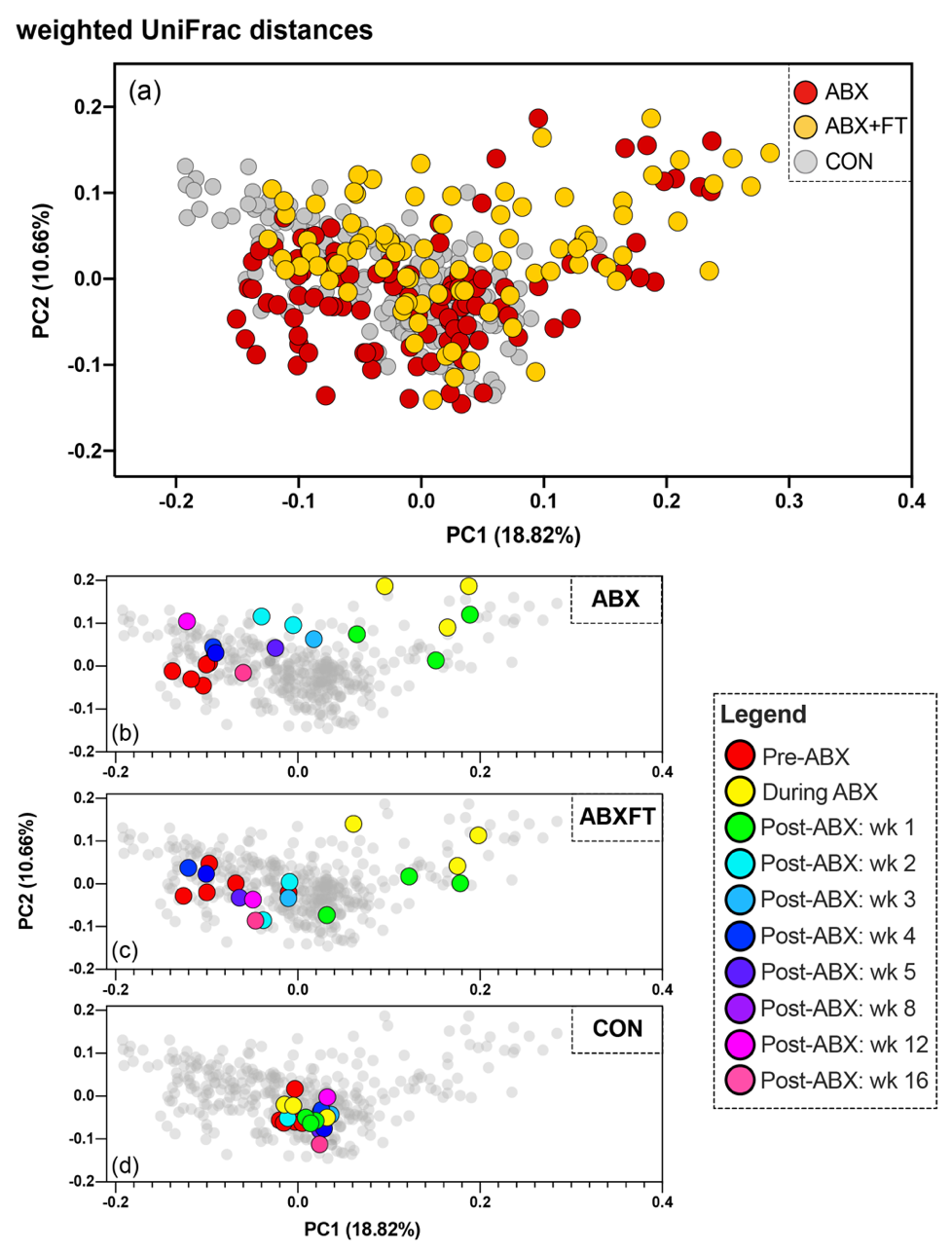


Figure S3. Principal coordinate analyses of weighted UniFrac beta diversity including (a) all samples colored by the three experimental groups: healthy, male ring-tailed lemurs (*Lemur catta*) that received no treatment (CON), antibiotics only (ABX), or antibiotics plus fecal transfaunation (ABXFT). (b, c, d) beta diversity of a representative animal from each of the three experiment groups with data points colored according time of collection (pre-, during, and post-antibiotic treatment; gray points are data from all other animals).

***iii. Supplementary tables of bacterial covariation***

Tables S1 and S2 present the mean correlation values for pairwise bacterial covariations in CON, ABX, and ABXFT animals. Correlation values were calculated using log-ratio abundances in a Bayesian multivariate Gaussian process (for more details, see main text Methods, *Statistical analyses* and Supplementary Materials, *i. Descriptions of statistical models*). Correlations with values of ⍴ > 0.5 or ⍴ < -0.5 were considered strong associations and reported in these tables.

Table S1. Pairwise bacterial associations with >-0.5 or <0.5 mean correlation values across the treatment and recovery periods.

| **Pairwise comparisons – During ABX treatment and recovery periods** | | **mean correlation values** | | | |
| --- | --- | --- | --- | --- | --- |
| **taxon 1** | **taxon 2** | **ABX** | **ABXFT** | **CON** |  |
| genus Bacteroides | genus Parabacteroides | 0.798 | 0.785 | - |  |
| genus Ruminococcaceae NK4A214 group | genus Ruminococcus 1 | 0.787 | - | - |  |
| genus Christensenellaceae R-7 group | genus Ruminococcaceae NK4A214 group | 0.665 | 0.766 | - |  |
| genus Ruminococcaceae NK4A214 group | family Erysipelotrichaceae | 0.618 | - | - |  |
| genus Blautia | genus Ruminococcaceae UCG-004 | 0.603 | - | - |  |
| genus Ruminococcaceae UCG-008 | genus Phascolarctobacterium | 0.597 | - | - |  |
| genus Roseburia | genus Ruminococcus 1 | 0.592 | - | - |  |
| genus Bacteroides | genus Barnesiella | 0.589 | 0.636 | - |  |
| genus Ruminococcus 1 | family Erysipelotrichaceae | 0.575 | - | - |  |
| genus Prevotellaceae NK3B31 group | genus Lachnospiraceae NK4A136 group | 0.574 | - | - |  |
| genus Cerasicoccus | order WCHB1-41 | 0.570 | 0.514 | 0.631 |  |
| genus Faecalibacterium | genus Ruminococcaceae NK4A214 group | 0.565 | - | - |  |
| genus Faecalibacterium | genus Ruminococcus 1 | 0.565 | - | - |  |
| genus Lachnospiraceae NK4A136 group | genus Ruminococcus 1 | 0.560 | - | - |  |
| genus Barnesiella | genus Desulfovibrio | 0.555 | - | - |  |
| genus Treponema 2 | family Bacteroidales RF16 group | 0.555 | - | - |  |
| genus Bacteroides | family Christensenellaceae | 0.551 | - | - |  |
| genus Bacteroides | family Clostridiales vadinBB60 group | 0.542 | - | - |  |
| genus Parabacteroides | genus Bilophila | 0.538 | 0.668 | - |  |
| genus Prevotellaceae NK3B31 group | genus Ruminococcaceae UCG-005 | 0.537 | - | - |  |
| genus Barnesiella | genus Parabacteroides | 0.531 | 0.607 | - |  |
| genus Sphaerochaeta | family Clostridiales vadinBB60 group | 0.524 | - | - |  |
| genus Parabacteroides | family Christensenellaceae | 0.520 | - | - |  |
| genus Christensenellaceae R-7 group | genus Ruminococcus 1 | 0.516 | - | - |  |
| genus Bacteroides | genus Bilophila | 0.515 | 0.645 | - |  |
| genus Bacteroides | genus UBA1819 | 0.514 | 0.620 | - |  |
| genus Angelakisella | genus Ruminiclostridium 5 | 0.514 | - | - |  |
| genus Roseburia | genus Ruminococcaceae NK4A214 group | 0.507 | - | - |  |
| genus Roseburia | genus Faecalibacterium | 0.501 | - | - |  |
| genus Ruminococcaceae UCG-008 | genus Other | -0.514 | - | - |  |
| genus Bacteroides | genus [Eubacterium] coprostanoligenes group | -0.534 | -0.557 | - |  |
| genus Bacteroides | genus Ruminococcaceae NK4A214 group | -0.540 | - | - |  |
| genus Bacteroides | genus Ruminococcus 1 | -0.542 | - | - |  |
| genus Parabacteroides | genus [Eubacterium] coprostanoligenes group | -0.551 | -0.527 | - |  |
| genus Bacteroides | genus Lachnospiraceae NK4A136 group | -0.555 | - | - |  |
| genus Ruminococcaceae NK4A214 group | genus Ruminococcaceae UCG-005 | - | 0.615 | - |  |
| genus Ruminococcaceae UCG-014 | family Lachnospiraceae | - | 0.544 | - |  |
| genus Bacteroides | genus Anaeroplasma | - | 0.540 | - |  |
| genus Parabacteroides | genus Coprococcus 1 | - | 0.529 | - |  |
| genus Barnesiella | genus Bilophila | - | 0.527 | - |  |
| genus Parabacteroides | genus UBA1819 | - | 0.519 | - |  |
| genus Candidatus Saccharimonas | family Lachnospiraceae | - | 0.511 | - |  |
| genus UBA1819 | genus Bilophila | - | 0.502 | - |  |
| genus Parabacteroides | genus Ruminococcaceae UCG-005 | - | -0.518 | - |  |
| genus Rikenellaceae RC9 gut group | genus Bilophila | - | -0.520 | - |  |
| genus Parabacteroides | genus Ruminococcaceae NK4A214 group | - | -0.530 | - |  |
| genus Prevotella 9 | genus Other | - | -0.533 | - |  |
| genus Rikenellaceae RC9 gut group | genus Parabacteroides | - | -0.548 | - |  |
| genus Bacteroides | genus Ruminococcaceae UCG-005 | - | -0.555 | - |  |
| genus Bacteroides | genus Rikenellaceae RC9 gut group | - | -0.561 | - |  |
| genus Bacteroides | family Lachnospiraceae | - | -0.569 | - |  |
| genus Ruminococcaceae UCG-014 | genus Bilophila | - | -0.570 | - |  |
| genus Ruminococcaceae UCG-014 | genus UBA1819 | - | -0.587 | - |  |
| genus Parabacteroides | family Lachnospiraceae | - | -0.606 | - |  |
| genus Parabacteroides | genus Ruminococcaceae UCG-014 | - | -0.624 | - |  |
| genus Bacteroides | genus Ruminococcaceae UCG-014 | - | -0.642 | - |  |

Table S2. Pairwise bacterial associations with >-0.5 or <0.5 mean correlation values across the recovery period.

| **Pairwise comparisons - Recovery period** | | **mean correlation values** | | |
| --- | --- | --- | --- | --- |
| **taxon 1** | **taxon 2** | **ABX** | **ABXFT** | **CON** |
| genus Bacteroides | genus Parabacteroides | 0.726 | 0.713 | - |
| genus Prevotellaceae NK3B31 group | genus Ruminococcaceae UCG-005 | 0.651 | - | - |
| genus Succinivibrio | genus Treponema 2 | 0.620 | - | - |
| genus Bacteroides | genus Angelakisella | 0.617 | - | - |
| genus Ruminiclostridium 5 | genus Bilophila | 0.606 | - | - |
| family Muribaculaceae | genus Prevotellaceae NK3B31 group | 0.552 | - | - |
| family Bacteroidales RF16 group | genus Treponema 2 | 0.543 | - | - |
| genus Blautia | genus Ruminococcaceae UCG-004 | 0.536 | - | - |
| genus Bacteroides | genus Anaeroplasma | 0.531 | - | - |
| genus Rikenellaceae RC9 gut group | genus [Eubacterium] coprostanoligenes group | 0.529 | - | - |
| genus Treponema 2 | genus Cerasicoccus | 0.528 | - | - |
| genus Parabacteroides | genus Angelakisella | 0.521 | - | - |
| genus Oscillospira | genus Ruminiclostridium 5 | 0.518 | - | - |
| genus Lachnoclostridium | genus Oribacterium | 0.513 | - | - |
| genus Christensenellaceae R-7 group | genus Ruminococcaceae NK4A214 group | 0.506 | 0.518 | - |
| genus Parabacteroides | genus Oscillospira | 0.502 | - | - |
| genus Blautia | genus GCA-900066575 | 0.501 | - | - |
| genus p-1088-a5 gut group | genus Treponema 2 | 0.500 | - | - |
| genus Prevotella 9 | genus Ruminiclostridium 5 | -0.521 | - | - |
| genus Parabacteroides | genus Sarcina | -0.536 | - | - |
| genus Bacteroides | genus Ruminococcaceae UCG-008 | - | 0.648 | - |
| order WCHB1-41 | genus Cerasicoccus | - | 0.629 | 0.568 |
| genus Butyricimonas | genus Prevotella 1 | - | 0.605 | - |
| genus Bacteroides | genus Roseburia | - | 0.595 | - |
| genus Oscillospira | genus Ruminococcaceae NK4A214 group | - | 0.592 | - |
| genus Parabacteroides | genus Coprococcus 1 | - | 0.576 | - |
| genus Ruminococcaceae NK4A214 group | genus Ruminococcaceae UCG-005 | - | 0.575 | - |
| genus Rikenellaceae RC9 gut group | genus Treponema 2 | - | 0.572 | - |
| genus Oscillospira | genus Ruminococcaceae UCG-005 | - | 0.566 | - |
| genus Parabacteroides | genus Bilophila | - | 0.561 | - |
| genus Bacteroides | genus Bilophila | - | 0.547 | - |
| genus Cerasicoccus | genus [Eubacterium] coprostanoligenes group | - | 0.534 | - |
| genus Parabacteroides | genus Lachnospiraceae UCG-001 | - | 0.533 | - |
| family Muribaculaceae | genus Ruminococcaceae UCG-013 | - | 0.514 | - |
| genus Bacteroides | genus Barnesiella | - | 0.513 | - |
| genus Prevotellaceae UCG-003 | genus Blautia | - | 0.513 | - |
| genus Roseburia | genus Ruminococcaceae UCG-008 | - | 0.512 | - |
| genus Bacteroides | genus Lachnospiraceae UCG-001 | - | 0.512 | - |
| genus Bacteroides | genus UBA1819 | - | 0.512 | - |
| family Muribaculaceae | genus Lachnospiraceae NK4A136 group | - | 0.511 | - |
| genus Lachnoclostridium | genus Ruminococcaceae UCG-008 | - | 0.506 | - |
| genus Ruminococcaceae UCG-014 | genus Bilophila | - | -0.509 | - |
| genus Bacteroides | family Lachnospiraceae | - | -0.515 | - |
| genus Bacteroides | genus [Eubacterium] coprostanoligenes group | - | -0.520 | - |
| genus Parabacteroides | family Lachnospiraceae | - | -0.534 | - |
| genus Lachnospiraceae UCG-001 | genus Ruminococcaceae UCG-014 | - | -0.542 | - |
| genus Parabacteroides | genus Ruminococcaceae UCG-014 | - | -0.551 | - |
| genus Bacteroides | genus Ruminococcaceae UCG-014 | - | -0.572 | - |
| genus Ruminococcaceae UCG-014 | genus UBA1819 | - | -0.597 | - |
| genus Cerasicoccus | order Rhodospirillales | - | - | 0.532 |
